# Homocysteine, Cortisol, Diabetes Mellitus, and Psychopathology

**DOI:** 10.1155/2015/354923

**Published:** 2015-02-01

**Authors:** K. Kontoangelos, C. C. Papageorgiou, A. E. Raptis, P. Tsiotra, V. Lambadiari, G. N. Papadimitriou, A. D. Rabavilas, G. Dimitriadis, S. A. Raptis

**Affiliations:** ^1^1st Department of Psychiatry, Athens University Medical School, Eginition Hospital, 11528 Athens, Greece; ^2^University Mental Health Research Institute, 11527 Athens, Greece; ^3^2nd Department of Internal Medicine, Research Institute and Diabetes Center, Athens University Medical School, Attikon University Hospital, 12462 Athens, Greece; ^4^Hellenic National Center for Research, Prevention and Treatment of Diabetes Mellitus and Its Complications (HNDC), 10675 Athens, Greece

## Abstract

*Objective*. This study investigates the association of homocysteine and cortisol with psychological factors in type 2 diabetic patients. *Method*. Homocysteine, cortisol, and psychological variables were analyzed from 131 diabetic patients. Psychological factors were assessed with the Eysenck Personality Questionnaire (EPQ), Hostility and Direction of Hostility Questionnaire (HDHQ), the Symptom Checklist 90-R (SCL 90-R), the Zung Self-Rating Depression Scale (ZDRS), and the Maudsley O-C Inventory Questionnaire (MOCI). Blood samples were taken by measuring homocysteine and cortisol in both subgroups during the initial phase of the study (*T*0). One year later (*T*1), the uncontrolled diabetic patients were reevaluated with the use of the same psychometric instruments and with an identical blood analysis. *Results*. The relation of psychoticism and homocysteine is positive among controlled diabetic patients (*P* value = 0.006 < 0.05) and negative among uncontrolled ones (*P* value = 0.137). Higher values of cortisol correspond to lower scores on extraversion subscale (*r*_*p*_ = −0.223, *P* value = 0.010). Controlled diabetic patients showed a statistically significant negative relationship between homocysteine and the act-out hostility subscale (*r*_sp_ = −0.247, *P* = 0.023). There is a statistically significant relationship between homocysteine and somatization (*r*_sp_ = −0.220, *P* = 0.043). *Conclusions*. These findings support the notion that homocysteine and cortisol are related to trait and state psychological factors in patients with diabetes mellitus type 2.

## 1. Introduction

In recent years, studies have shown that elevated levels of homocysteine in the blood are an important and independent risk factor for cardiovascular disease, as well as for vascular dementia and Alzheimer's disease [1–3]. The literature indicates that chronic alcoholism leads to hyperhomocysteinaemia [4].

The concept of vascular depression, proposed by Alexopoulos and colleagues [5], assumes that many elderly people have a medical history of vascular disease associated with predisposing aggravating factors such as diabetes, smoking, hypercholesterolaemia, hypertriglyceridemia, and elevated homocysteine. A study by Almeida et al. demonstrated a high correlation between depression and high homocysteine values [6]. Homocysteine was also associated with hostility when the sample was controlled for age, sex, educational level, and dietary habits. Regarding the direction of hostility, extroverted patients' scores had a greater correlation with high homocysteine levels compared to introverted patients [7]. Microangiopathy has a high incidence among type 2 diabetic patients, with an early onset and rapid progression compared with nondiabetic patients. Homocysteine may be a determining risk factor for the development of microangiopathy in patients with type 2 diabetes [8]. The plasma concentrations of homocysteine in patients with type 2 diabetes are higher than those of healthy patients.

Obese patients with type 2 diabetes presented higher homocysteine values when compared to healthy controls. Additionally, underweight patients with type 2 diabetes had lower homocysteine values compared to healthy controls [9].

A study by Hickie et al. [10] explored the relationship between type 2 diabetes and depression. Specifically, they examined the development of white matter lesions in periventricular and subcortical hyperintensities in patients with depression. The study compared risk factors (diabetes, hypertension, and elevated homocysteine levels) in depressed patients separately for each aggravating cardiovascular factor.

Cortisol is a product of the biosynthetic activity of the adrenal cortex. The stimulus for its secretion is given by ACTH, which is secreted after stimulation of the adenohypophysis by hypothalamic CRF. Disturbances in cortisol values are observed during changes in the levels of ACTH, depression, psychological stress, for example, hypoglycaemia, fever, trauma, surgery, fear, and pain. Cortisol interacts with other chemical substances and affects the production of insulin by increasing gluconeogenesis [11]. Many clinical signs confirm the involvement of the hypothalamus pituitary adrenal axis in depression.

Approximately 50% of depressed patients have increased cortisol values. This probably involves the increased secretion of hypothalamic CRH resulting in an increase of ACTH and cortisol [12].

The literature indicates the dysfunction of the adrenergic system and the 5-HT (5-hydroxytryptamine receptors) system, combined with hyperactivity of the neurons that use the CRF agent, as a major cause of depressive and anxiety symptoms. High values of the corticotropin releasing factor (CRF) have been found in the CSF of patients with depression [13].

Clinical forms of Cushing's syndrome are present in some patients with major depressive episodes, despite the fact that cortisol levels are lower in depression than in Cushing's syndrome. However, only half of patients with depression have elevated cortisol values. For example, lack of sleep is associated with a transient increase in cortisol, in combination with impaired glucose tolerance [14].

In type 2 diabetes, insulin resistance is characterized by impaired response to insulin action in relation to the metabolism of carbohydrates, which is considered one of the main atherosclerotic risk factors. Several studies indicate the relationship between major depression and insulin resistance. One of the pathophysiological mechanisms, correlating insulin resistance with depression, is the hyperactivity of the hypothalamic-pituitary-adrenal axis [15].

Diabetes mellitus is common in patients with Cushing's syndrome. This syndrome affects glucose tolerance and may cause insulin resistance at a biochemical, triggering type 2 diabetes. However, elevated cortisol levels may be associated with type 1 diabetes. Bruehl et al. studied the action of cortisol in patients with type 2 diabetes in relation to hippocampus atrophy. It was observed that patients with type 2 diabetes showed reduced cortisol activity, in combination with reduced hippocampal volume [16].

The incidence of diabetes mellitus is greater in patients with schizophrenia than in the general population. Antipsychotic drugs have been related to the development of diabetes. In addition, patients with schizophrenia are often exposed to stressful conditions. These conditions lead to hyperactivity of the HPA axis, caused by the increased production of cortisol and adrenaline. Both these hormones play an important role in the onset of diabetes in patients with schizophrenia [17]. Considering the above, the aims of the present study was to explore the relationship between homocysteine, cortisol, and type 2 diabetes mellitus with psychological factors and specifically the direction of the relationship between obsessive compulsive symptomatology, depression, somatization, and personality with the above biochemical parameters and diabetes control. Secondly, how these relations are influenced when the diabetic patients improve their metabolic profile.

## 2. Material and Methods

The present study was conducted at the Attikon University Hospital and the sample was randomly selected from the Diabetes Centre of the 2nd Internal Medicine-Propaedeutic Department, Research Institute. This is the third part of a study that consisted of the identification of neuropsychological and neuroendocrinological factors of diabetes mellitus type 2. The first part examined the relationship between obsessive compulsive disorder and depression in diabetic patient [18] while the second part examined the relation between oxytocin, somatization, and personality traits [19]. All the patients had a diagnosis of type 2 diabetes mellitus. The study excluded patients with malignant diseases, endocrinological syndromes, known severe peripheral arteriopathy, coronary disease, and kidney failure, patients with amputations and current psychopathology, and those that were under treatment with antidepressants, tranquillizers, antipsychotics, and anticonvulsant medication. The Ethics Committee of Attikon University Hospital approved the study and all participants were informed of its purposes and gave their written consent. The sample consisted of 131 diabetic patients, 76 males and 55 females. The demographic characteristics of the sample were provided in Table 1.

Patients were divided into two groups according to the level of diabetes control as indicated by glycosylated haemoglobin (HbA1c): group A consisted of 86 controlled diabetic patients (HbA1c < 7) and group B of 45 uncontrolled diabetic patients (HbA1c ≥ 7). Group A consisted of 36 female and 50 male participants, while group B of 20 female and 25 male participants, respectively. During the initial phase of the study (*T*0), blood samples were taken to measure homocysteine and cortisol serum levels. One year later (*T*1), the uncontrolled diabetic patients were reevaluated with the use of the same psychometric instruments and with identical blood analysis. During the intermediate year, an intensive effort to improve their metabolic profile was performed with frequent appointments with the hospital dieticians and diabetologists and adjustments of their medications.

In this year, an intensive effort to improve their metabolic profile was performed with more frequent appointments at the Diabetes Centre, adjusting their medication and diet. From the initial sample of 45 uncontrolled diabetic patients, 4 died from natural causes, while 10 withdrew from the study for other personal and family reasons, such as relocation. Finally, 31 uncontrolled diabetic patients were reassessed. The reevaluated sample consisted of 17 male and 14 female participants. Ten of these 31 uncontrolled patients had already managed to improve their metabolic profile (HbA1c < 7) in the preceding year between Phase 1 (*T*0) and Phase 2 (*T*1) of the study.

During the initial evaluation, all the participants were assessed with the following psychometric questionnaires.

(A) Psychometric Personality scale of extraversion, neuroticism, psychoticism (Eysenck Personality Questionnaire, EPQ) [20]: the Eysenck Personality Questionnaire consists of 84 entries evaluated by the patient with a yes or no. The purpose of this questionnaire is to explore four dimensions of personality: psychoticism (P), neuroticism (N) extraversion (E), and lying (L). Scales N and L are of particular clinical interest. The N scale is the best studied and is associated with a clinical diagnosis of neurosis or oral personalities according to psychoanalytic terminology. The E scale corresponds roughly to histrionic personalities. P scale corresponds to obsessive-compulsive personalities and is unrelated to psychosis. Finally, L scale controls the degree of hypocrisy of the examined party but can also be high in patients with psychosomatic disorders who are not pretending. A weighted Greek version is available [21].

(B) Psychometric Hostility and Direction of Hostility Questionnaire (HDHQ) [22]: the Psychometric Scale of Hostility and Direction of Hostility consists of 51 entries which is self-completed. It consists of four subscales: (a) intropunitiveness, (b) extrapunitiveness, (c) direction of hostility, and (d) general hostility. The assessment instrument consists of five subscales: (a) the urge to act out hostility, (b) criticism of others, (c) projected delusional or paranoid hostility, (d) self-criticism, and (e) delusional guilt. The first three subscales are summed to form an extrapunitive score and the other two are summed to yield an intropunitive score.

(C) Brief symptom Inventory (SCL-90) [23]: the SCL-90 questionnaire is self-completed and measures 9 psychopathology parameters (as many as its subscales), which are (1) somatization, (2) depression, (3) anxiety, (4) phobic anxiety, (5) obsessive compulsive, (6) paranoid ideation, (7) psychoticism, (8) hostility, and (9) interpersonal sensitivity. The questionnaire includes 90 questions in total. All entries are rated from 0 to 4, giving a total score of 360. The scale is used to extrapolate 3 aggregate indexes: (a) the general severity index, (b) the positive symptoms distress index, and (c) the positive symptoms total. A weighted Greek version is available [24].

(D) The Maudsley O-C Inventory Questionnaire (MOCI) [25]: obsessive-compulsive symptoms are measured by using the Maudsley Obsessional Compulsive Inventory (MOCI). In our investigation, the focus was on the types of obsessive-compulsive symptomatology and not on obsessional personality traits. It appears that people who suffer from observable obsessive-compulsive rituals complain about 4 main types of problems which are labelled: checking, cleaning, slowness, and doubting. These labels refer to types of problems and not to types of people and a person may suffer from more than one type. “Cleaning” describes clear-cut complaint, which is noted in most psychiatric descriptions of obsessional disorders. Those who obtain a high slowness score tend not to suffer from persistent unpleasant thoughts. From our clinical experience, this is a characteristic of patients with obsessions, who suffer from slow repetitive behaviour with little or no anxiety and no persistent ruminations. They appear to be complaining of a type of ritual, which has been called “functionally autonomous,” the probable basis for “primary obsessional slowness.” Doubting is the resulting behavioural trait, which appears to describe a type of obsessional problem, often in clinical practice. A person suffering from this complaint is never certain that he has performed a ritual properly and often has serious doubts about simple daily activities. The individual possesses stern conscience, and this results in feelings of anguish [26].

(E) The Zung Self-Rating Depression Scale (ZDRS) [27]: Fountoulakis et al. (2001) introduced the Greek translation of the Zung Scale, where they proposed five factors to explain the severity of depression. The first factor reflects anxiety-depression, the second is the thought content factor, the third describes gastroenterological problems, the fourth represents irritability, and the fifth factor summarizes information about social functioning [28].

### 2.1. Statistical Analysis

Linear relationships between continuous variables were measured by the Pearson correlation coefficients for normally distributed variables or the Spearman correlation coefficients otherwise. Associations between groups of patients and continuous variables were explored through the Students' *t*-test under the assumption of normally distributed variables and equality of variances. In the case of small sample sizes or asymmetric distributions, the corresponding nonparametric tests were performed, such as Mann-Whitney test for *t*-test and Wilcoxon test for the paired *t*-test. Scales based on the sum of binary questions (such as the scales of EPQ, HDHQ, and MOCI) are assumed to follow the binomial distribution and thus binomial generalized linear models were used to explore the corresponding relations. Over dispersion, problems were handled by using the quasibinomial distribution family. The subscales of SCL-90 scale are rescaled in order to lie in the interval between 0 and 1 and, therefore, zero or one inflated beta regression analysis is used. Multiple logistic regression analysis was performed to examine the association between the metabolic profile, homocysteine, cortisol, and binary Zung Scales. The statistical tests presented here are two-tailed and compared with the statistical level of 5%.

The statistical package SPSS 21 was used to perform descriptive analysis, *t*-test, linear correlation, and logistic regression analysis. The statistical package GAMLSS (Rigby R.A.2005) in R 2.15.1 was used to perform beta regression analysis. Binomial regression analysis was performed on SPSS. However, glm package in R was also used to handle overdispersion problems. Descriptive analysis presents mean and standard deviation for scale variables and counts and percentages for categorical variables.

## 3. Results

### 3.1. First Evaluation

#### 3.1.1. EPQ

The relationships of the biochemical parameters of homocysteine and cortisol with the subscales of the personality questionnaire EPQ were examined with respect to the metabolic profile. Since the EPQ questionnaire consists of polar questions (yes/no), each subscale is assumed to follow the binomial distribution with number of trials equal to the number of questions (24 for psychoticism, 22 for neuroticism, and 19 for extraversion and lie). Therefore, we use binomial generalized linear models to examine the corresponding relations.

**Figure 1:**
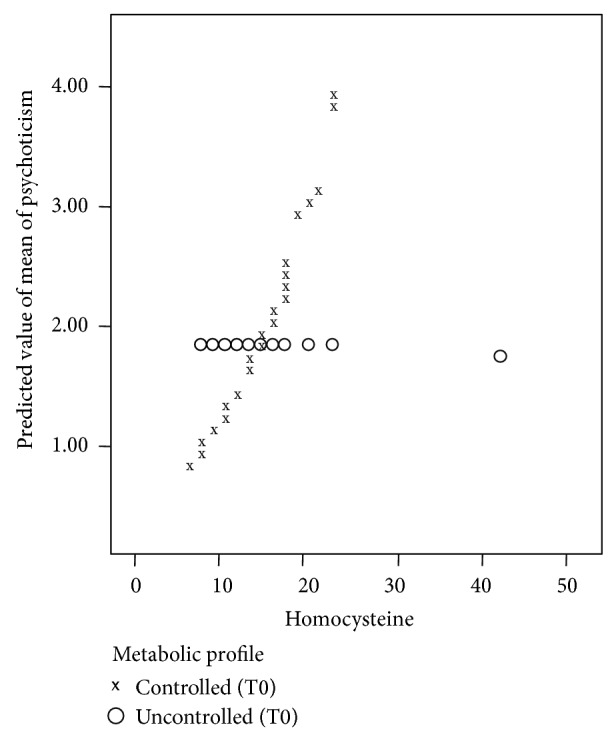
Predicted values of mean of psychoticism by homocysteine levels and diabetes profile.

The relation of psychoticism and homocysteine is positive among controlled (*P* value = 0.006 < 0.05) and negative among uncontrolled diabetic patients (*P* value = 0.137). Binomial generalized linear model verifies the significance of this relation, where the effect of the interaction term (homocysteine × metabolic profile) is significant.  Figure 1 shows the predicted values of mean of the psychoticism scale against the values of homocysteine.

It turns out that there is significant correlation between the subscale of extraversion and cortisol. Analysis of covariance shows that this relation does not differ among the controlled and uncontrolled patients (both effect of metabolic profile and interaction term are not significant). We therefore present the Pearson correlation of the entire sample which is (*r*_*p*_ = −0.223) (*P* value = 0.010) (Table 2). Higher values of cortisol correspond to lower scores on extraversion subscale.

#### 3.1.2. HDHQ

Further analysis examining the existence of relationships between the biochemical parameters and the overall hostility scale and its subscales (HDHQ) was performed. The scores of controlled diabetic patients showed a statistically significant negative relationship between homocysteine and the act-out hostility subscale (*r*_sp_ = −0.247, *P* = 0.023), indicating a negative relationship between these biochemical parameters and the hostility subscale. Controlled patients with higher homocysteine values are expected to score lower on the hostility subscale.

The questions in HDHQ questionnaire have two possible answers (yes/no) and thus each subscale is assumed to follow the binomial distribution with number of trials equal to the number of questions (13 for act-out hostility, 12 for criticism of others, 9 for paranoid hostility, 11 for self-criticism, and 7 for delusional guilt). Binomial generalized linear models are used to examine the relation of the subscales of HDHQ with the biochemical parameters and metabolic profile.

Table 3 presents the results when fitting the binomial generalized linear model on the scale of act-out hostility. The relation of this scale with homocysteine and metabolic profile is examined controlling for age, gender, triglyceride, and the interaction of triglyceride with metabolic profile. Patients with higher levels of homocysteine are expected to have lower scores on the scale of act-out hostility given that they have the same metabolic profile, gender, age, and triglyceride levels. Uncontrolled patients are expected to have higher scores on this scale than controlled diabetes patients, given that they have the same levels of homocysteine, gender, age, and triglyceride levels.

Besides that, uncontrolled patients have higher scores on the scale of delusional guilt than controlled diabetic patients given that they have the same levels of homocysteine, gender, and age.

Patients who are intropunitive have higher levels of cortisol than extrapunitive patients. The difference is statistical significant (*P* value = 0.013).

#### 3.1.3. SCL-90

We examine the relationship between the SCL-90 scale, homocysteine, and cortisol separately for controlled and uncontrolled patients. There is a statistically significant relationship between homocysteine and somatization (*r*_sp_ = −0.220, *P* = 0.043), characterized by a negative relation which means that patients with higher homocysteine values will score lower on the somatization scale. There was a positive statistically significant correlation between cortisol and phobic anxiety (*r*_sp_ = 0.215, *P* = 0.047), which means that controlled patients with higher cortisol values are expected to score higher on the scale of phobic anxiety. There were no statistically significant relationships for uncontrolled diabetic patients.

**Figure 2:**
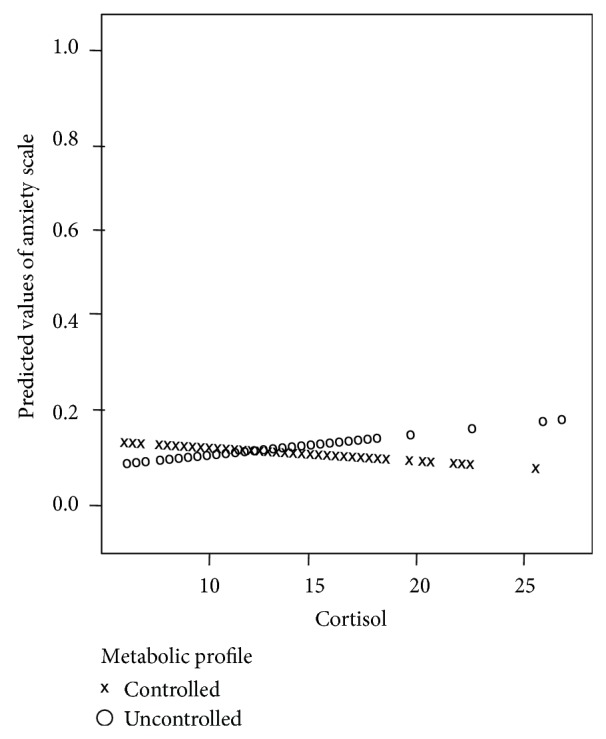
Predicted values of anxiety scale by cortisol and metabolic profile.

The relations among the subscales of SCL-90 and homocysteine or cortisol were further examined using zero-inflated beta regression.  Figure 2 shows graphically the relation between anxiety, cortisol, and metabolic profile, where the anxiety scale and cortisol have positive relation among uncontrolled patients and slightly negative relation among controlled patients.  Table 4 presents the estimates of zero-inflated beta distribution's parameters. The location parameter (*μ*) depends on cortisol, metabolic profile, interaction of cortisol and metabolic profile, age, and gender, whereas scale and shape parameters are treated as fixed ones.

#### 3.1.4. MOCI

Examining the relationship between homocysteine, cortisol, and the MOCI scale, there was a statistically significant relationship between the values of homocysteine and the cleaning subscale (*r*_*p*_ = 0.325, *P* = 0.029) in uncontrolled diabetic patients, whereas no significant relationships were found between the biochemical parameters and the subscales of the MOCI for controlled diabetic patients.

**Figure 3:**
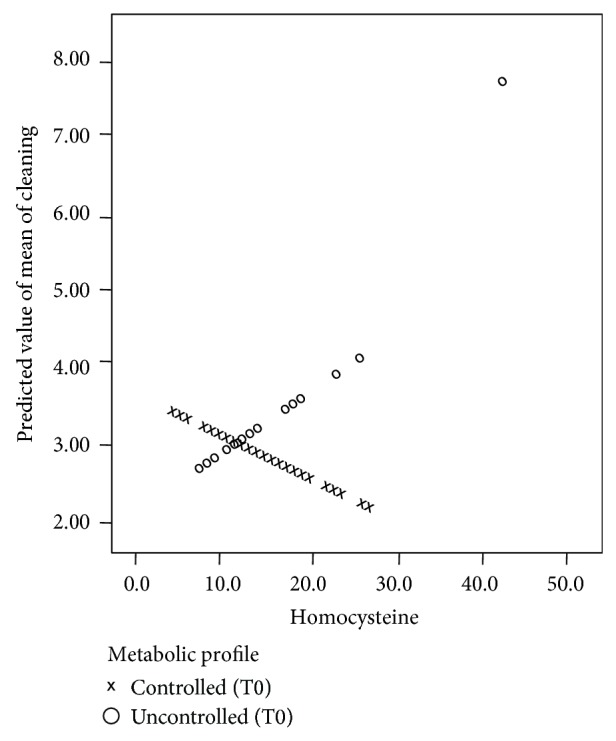
Predicted values of mean of cleaning by homocysteine levels and metabolic profile.

Binomial generalized linear models are used to further examine the aforementioned relations. The subscales of MOCI scale are the response variables with number of trials equal to 8 for checking, 11 for cleaning, 8 for slowness, and 7 for doubting. A significant relation is found between the cleaning subscale and the homocysteine levels and diabetes profile (Table 5).  Figure 3 presents this relation graphically. Uncontrolled patients with higher values of homocysteine have higher probability of giving positive answers on the questions related to the subscale of cleaning. On the other hand, controlled patients with higher values of homocysteine have lower chance of giving positive answers on these questions.

#### 3.1.5. Zung

The relationship of the biochemical parameters of cortisol and homocysteine with the Zung Scale was explored by converting the 20 questions of the scale into binary variables. When the patient's response was “a little of the time,” this was categorized as 0, while all other answers were grouped in the same category taking the value 1 (some of the time or more frequently). The purpose of this categorization was to examine the existence of a relationship between the patients' responses, their metabolic profile, and the biochemical parameters.

The scores of uncontrolled diabetic patients showed a statistically significant relationship between libido and homocysteine. The more decreased the libido patients experienced, the higher the values of homocysteine measured (11.16 ± 2.04 versus 15.21 ± 6.17) (*P* = 0.002).

Logistic regression analysis is used to examine the relation of Zung subscales with respect to the metabolic profile and the biochemical parameters (Table 6). Subscales are significantly related to the diabetes group and the homocysteine levels (the interaction term is significant) when controlling for gender and age.

**Figure 4:**
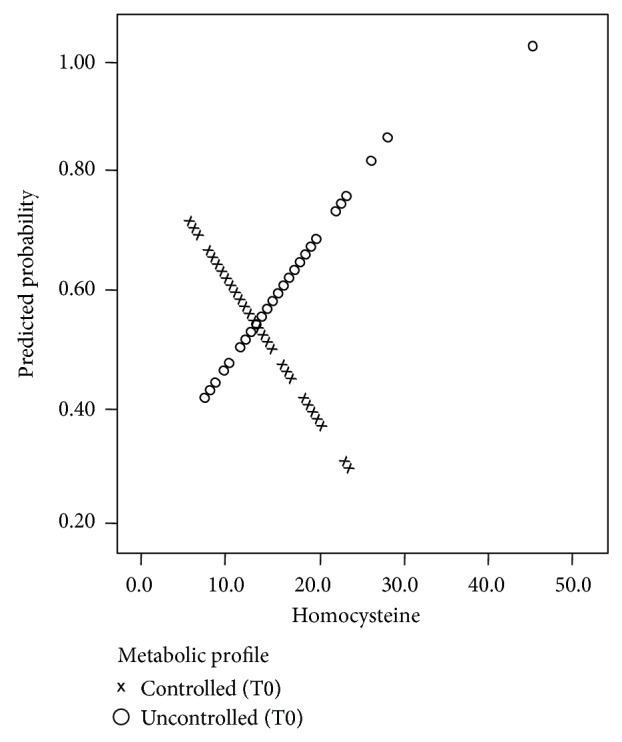
Predicted probability of frequent fatigue by homocysteine levels and metabolic profile.

Figure 4 presents the predicted probability of feeling tired frequently with respect to the levels of homocysteine and diabetes profile. It turns out that as the levels of homocysteine increase, the predicted probability of frequent fatigue increases for the uncontrolled diabetes patients, whereas it decreases for the controlled patients.

Both controlled and uncontrolled patients have a decreased libido as the levels of homocysteine increase; however, the probability increases more rapidly in uncontrolled than controlled patients.

Table 7 displays the subscales that are related significantly to cortisol and the diabetes profile (the interaction term is significant) when controlling for gender. Uncontrolled diabetic patients have frequent sleeping problems when they have higher levels of cortisol, whereas controlled patients have less probability of sleeping problems when the cortisol levels increase.

Concerning the patients' libido, controlled patients have a higher probability of decreased libido as the levels of cortisol increase, while uncontrolled patients are less likely to have decreased libido as the levels of cortisol increase.

### 3.2. Second Assessment, T1

We investigated whether the values of the biochemical parameters and psychometric tests differed between the uncontrolled patients of the first evaluation and the controlled patients of the second evaluation. We used a paired test to conclude on average whether the levels of the biochemical parameters and psychometric tests differed in the 10 individuals found to have achieved control at the second evaluation.

We observed that the values of cortisol dropped when the patients managed to control their metabolic profile, while homocysteine values increased when patients reduced the levels of HbA1c below 7. However, the changes of these biochemical parameters, during the first and second evaluation, do not appear to be statistically significant (0.51 ± 0.23 versus 0.87 ± 0.46, *P* = 0.042).

Similarly, we checked whether the scores of the psychometric tests differed significantly between the first and second evaluation. The scores of psychoticism, neuroticism, and lying declined when uncontrolled diabetic patients achieved control, while the extroversion score increased.

Act-out hostility and the direction of hostility scores increased when HbA1c values fell below the threshold of 7, while the total hostility index, as well as all other scales, dropped when patients controlled their metabolic profile. The reduction of the total index of hostility, of criticism of others, and of external hostility appears to be statistically significant (*P* = 0.032, *P* = 0.011, and *P* = 0.038).

In addition, we examined whether the mean values of the subscales of SCL-90 changed significantly when patients regulated their metabolic profile. The scale of somatization did not change between the first and second evaluation, while the scale of anger-hostility increased. The remaining scales were reduced when HbA1c values fell below 7. However, the scales with a statistically significant decrease were the subscales of interpersonal sensitivity and psychoticism (*P* = 0.028 and *P* = 0.024).

Finally, we examined whether the mean values of the MOCI and Zung subscales changed significantly when patients regulated their metabolic profile. The Zung Scale did not change between the first and second evaluation. The scores of the subscales of thought content and irritability increased between the first and second evaluation, while the rest decreased. In the MOCI scale, the subscales of slowness and doubting did not change between the first and second evaluation. The control scale dropped, while the subscale of doubting increased between the first and second evaluation.

However, the differences in the mean values of the Zung and Maudsley scales and their subscales during the first and second evaluation did not appear to be statistically significant.

## 4. Discussion

We explored the relationship between homocysteine and psychometric tools. The finding revealed that, in controlled diabetic patients, high homocysteine values are associated with high psychoticism scores on the EPQ scale. Jensen et al. (1998) [29] studied 109 elderly patients using the EPQ scale and measuring the levels of homocysteine. The results show that plasma homocysteine concentrations greater than 15 *μ*mol/L in octogenarians were associated with worse subjective and objective health, poorer life satisfactions, restlessness, and feeling depressed. Authors found no correlation between elevated homocysteine values and the EPQ subscales (extraversion, neuroticism, and psychoticism).

Schizophrenia patients are characterized by elevated homocysteine level [30]. Most interestingly, this association is also present in first-episode schizophrenia patients, who are drug-naïve or minimally exposed to antipsychotics [31, 32]. Moreover, there is a growing body of evidence that one-carbon metabolism dysfunction along with increased homocysteine level might be implicated in the development of comorbid metabolic syndrome in this group of patients [33]. Homocysteine has now implicated in increased oxidative stress, DNA damage, the triggering of apoptosis, and excitotoxicity, all important mechanisms in neurodegeneration. The brain may be particularly vulnerable to high levels of homocysteine in the blood because it lacks two major metabolic pathways for its elimination: betaine remethylation and transsulfuration [34].

Furthermore, controlled diabetes patients with higher homocysteine values have low scores on the act-out hostility subscale of the HDHQ. This agrees with another study in which the authors studied 410 individuals of the overall population and showed a negative correlation between homocysteine values and act-out hostility [7]. Controlled diabetic patients with higher cortisol values have lower scores on the subscale of act-out hostility. We also found a statistically significant correlation between homocysteine and act-out hostility on our male controlled diabetic participants. Our results reveal a significant positive relationship between homocysteine and the direction of hostility subscale of the HDHQ in controlled diabetic patients.

The controlled diabetic patients with high levels of homocysteine scored low on the somatization scale of the SCL-90. In a study of 205 obese patients, Marchesini et al. (2002) showed no statistically significant association between homocysteine and the somatization scale of the SCL-90 [9].

Homocysteine levels are positively correlated with the cleaning subscale of the MOCI. In a study of 23 patients diagnosed with OCD according to DSM-IV, Atmaca et al. (2005) found elevated levels of homocysteine in patients with OCD compared to controls, while homocysteine levels were positively correlated with the scores of the Y-VOCKS scale [35]. Existing literature indicates that OCD may be the result of an ongoing pathological process associated with disturbances in serotonergic and dopaminergic neurons, which are also related to the pathophysiology of depression. OCD often entails depressive symptoms and coexists with depression. Obsessions and compulsions may be present in patients with depressive disorder [36].

Finally, homocysteine increases in uncontrolled diabetic patients, who often exhibit a decreased libido in the Zung Scale. Increased homocysteine values often accompany uncontrolled type 2 diabetes [37]. The concept of vascular depression, proposed by Alexopoulos and colleagues, assumes that many elderly people present a medical history of vascular disease associated with predisposing aggravating factors such as diabetes, smoking, hypercholesterolaemia, hypertriglyceridemia, and elevated homocysteine values [5].

A study by Almeida et al. (2007) reported a strong correlation between depression and high homocysteine values. Several risk factors for cardiovascular disease have also been associated with depression in older people: diabetes and insulin resistance, smoking abnormal lipid profile, and high plasma homocysteine. The association between depression and high total plasma homocysteine may be a consequence if the direct cardiovascular and neurotoxic effects of homocysteine as well as an indirect result of dysfunction of the methionine metabolism loop. Methionine is the precursor of S-adenosylmethionine, which is the methyl group donor for the synthesis and metabolism of several monoamines such as serotonin, dopamine, and noradrenaline. These metabolites play an important role in the pathogenesis of depression. Because homocysteine values are often elevated in elderly people, the PAF ratio (fraction of population to risk factor) was higher for homocysteine in relation to depression than other risk factors when associated with depression. This could be related to the hypothesis that the reduction of high homocysteine values reduces the prevalence of depression in elderly men [6].

When checking the relationship of cortisol and psychometric tools, we found that uncontrolled diabetic patients with high cortisol values score lower on the extraversion subscale of the EPQ scale. This is contrary to the study of Le Blanc and Ducharme which, in a study of 20 people, found that cortisol correlated positively with extraversion [38].

In addition, controlled diabetic patients with higher cortisol values have lower scores on the external hostility subscale of the HDHQ scale.

Pope and Smith (1991) studied hostility in 39 men, taking cortisol levels into account, based on the original hypothesis that cortisol is not an adrenal hormone that determines the physiological activity and stress but may be associated with atherosclerotic damage. The results showed that high levels of cortisol are associated with increased levels of hostility [39].

Cortisol in controlled diabetic patients is increased when patients experience intense phobic anxiety in the scale of the SCL-90. Cortisol is a glucocorticoid produced by the zona fasciculata of the adrenal cortex and, like other hormones of the adrenal cortex, is a derivative of cholesterol. Stress, either psychological or due to other reasons (trauma, surgery, injection, anaesthesia, and hypoglycaemia), and depression stimulate the releasing hormone of the adrenal cortex of hypothalamic CRH in the brain that leads to the production of ACTH by the pituitary gland, which acts on the adrenal glands by stimulating the production of hormones, which include cortisol, by the adrenal cortex [40].

The study by Augner and Hacker (2009) reached similar conclusions, reporting a positive correlation between cortisol and the subscales of the SCL-90 regarding somatization, phobic anxiety, depression, and OCD behaviour in 75 patients exposed to electromagnetic fields of mobile telephony [41]. Given that stress and glucocorticoids can cause damage to the prefrontal cortex, a consequence may be depression, anger, and anxiety [42]. The above research confirms our original aim and working hypothesis on the interaction of neuroendocrine and psychoimmunological factors with psychological-psychopathological parameters. Through statistical correlations, we extrapolate important conclusions for understanding parameters that are related both to the treatment and to the pathogenesis of diabetes mellitus.

In addition, the correlation of biochemical parameters with psychometric variables in the clinical setting could provide the clinical diabetologists with great help in evaluating potential psychopathological conditions that are undermined and revealed through the endocrinological-immunological profile of the diabetic patient.

In conclusion, the present results provide evidence that homocysteine and cortisol are closely related to the course and treatment of type 2 diabetes. Although the specific implications of the research on clinical practice remain uncertain, it is likely that additional work in this area will further demystify and validate essential mechanisms involved in the pathogenesis of type 2 diabetes. Such an approach also promises to help in treatment selection by providing an enhanced vocabulary for discussing concepts central to the treatment of diabetes.

## 5. Limitations

The limitations of this study are as follows.

1. The number of participants was small during the second evaluation, which was 45 uncontrolled diabetic patients at baseline assessment. Finally, we evaluated 31 uncontrolled diabetic patients of whom 10 were able to achieve control.
2. This is a sectional study in a specific period and a prospective study would be desirable. It probably makes sense for the research strategies to be perspective rather than sectional, by inclusion of the relevant genetic variables. A prospective study promises to also determine additional relevant parameters (cost, quality of life, and pain). In recent years, the understanding of the pathogenetic contribution of psychosocial factors in diabetic morbidity/mortality has increased substantially. On the contrary, the development of effective therapeutic interventions to change pathological lifestyles/behaviour and to reduce their impact remains a life challenge. The compliance of the patient with the therapeutic regimen is a fundamental problem. The issues raised for investigation: apart from depression, which other psychosocial variables have causative significance?
3. If other variables are involved, is there a one-way interaction or should we expect “synergies”?
4. What is the importance of the chronicity of stressful conditions?
5. It is concerned with a subset of the spectrum of psychopathology-personality.
6. It assumes the coevaluation of intraphenotypic and genotypic variables.

## 6. Conclusions

Homocysteine is related positively to psychoticism and negatively to the act-out hostility and somatization in controlled diabetic patients.

Cortisol is correlated negatively with extraversion and positively with phobic anxiety in controlled diabetic patients.

Homocysteine and cortisol are related to trait and state psychological factors in patients with diabetes mellitus type 2.

Control of diabetes depends on psychopathological data (added).

Biochemical parameters could be determinant factor for control of diabetes mellitus.

## Figures and Tables

**Table 1:** Demographic characteristics of the sample.

	Total (*n* = 131)		Controlled (*n* = 86)		Uncontrolled (*n* = 45)		*P*
	mean	±s.d.	mean	±s.d.	mean	±s.d.	
Age	64.0	±9.95	64.2	±10.94	63.6	±7.84	0.759
Duration	11.9	±8.73	11.5	±8.96	12.6	±8.31	0.498
Homocysteine	13.3	±4.37	12.9	±3.65	13.9	±5.49	0.278
Cortisol	13.6	±4.52	13.5	±4.46	13.7	±4.69	0.790

	*n*	%	*n*	%	*n*	%	

Education							
Elementary	106	80.9	71	82.6	35	77.8	0.791
Secondary	8	6.1	5	5.8	3	6.7	
Highest	17	13.0	10	11.6	7	15.6	
Occupation							
Housekeeping	49	37.4	30	34.9	19	42.2	0.686
Pensioners	40	30.5	26	30.2	14	31.1	
Civil servants	10	7.6	8	9.3	2	4.4	
Self-employed	32	24.4	22	25.6	10	22.2	

Parentheses display the standard deviations.

**Table 2:** Estimates of the binomial generalized linear model for the psychoticism scale of EPQ.

	Psychoticism	
	OR	*P*
Homocysteine	1.13	<0.001
Diabetes profile	1.02	0.897
Interaction of group and Homocysteine	0.88	0.003

Controlled for age and gender		

**Table 3:** Estimates of the binomial generalized linear model for the subscales of HDHQ.

	Act-out hostility		Delusional guilt	
	OR	*P*	OR	*P*
Homocysteine	0.95	0.010	0.97	0.263
Diabetes profile	1.30	0.040	1.41	0.029

	Controlled for gender, age, triglyceride, and interaction of triglyceride and metabolic profile		Controlled for gender and age	

**Table 4:** Estimates of location parameter.

*μ*	Anxiety	
	Estimates	*P*
Cortisol	−0.004	0.808
Diabetes profile	0.363	0.009
Interaction of cortisol and diabetes profile	0.062	0.025

Controlled for gender and age		

**Table 5:** Estimates of the binomial generalized linear model for the cleaning scale of MOCI.

	Cleaning	
	OR	*P*
Homocysteine	0.97	0.277
Metabolic profile	1.34	0.084
Interaction of homocysteine and diabetes profile	1.09	0.028

**Table 6:** Estimates of the logistic regression analysis on Zung subscales.

	Fatigue		Libido	
	OR	*P*	OR	*P*
Homocysteine	0.96	0.549	1.00	0.972
Metabolic profile	1.72	0.192	2.72	0.108
Interaction of homocysteine and metabolic profile	1.33	0.034	1.67	0.026

**Table 7:** Estimates of the logistic regression analysis on Zung subscales.

	Sleep		Libido	
	OR	*P*	OR	*P*
Metabolic profile	1.72	0.167	1.82	0.203
Cortisol	0.95	0.335	1.11	0.087
Interaction of cortisol and metabolic profile	1.21	0.049	0.82	0.042
